# Characterisation of the complete mitochondrial genome of *Bactrocera ruiliensis* (Diptera: Tephritidae), with its phylogenetic analysis

**DOI:** 10.1080/23802359.2019.1681320

**Published:** 2019-10-24

**Authors:** Yan-Ling Ren, Tao Wang, Maofa Yang, Weizhi Shao

**Affiliations:** a Guizhou Light Industry Technical College, Guiyang, P. R. China;; b Institute of Entomology, Guizhou University, Guiyang, P.R. China;; c Ruili Customs Integrated Technology Center, Ruili, P. R. China

**Keywords:** Mitogenome, Dacinae, *Bactrocera ruiliensis*, phylogeny

## Abstract

The complete mitochondrial genome (mitogenome) of the *Bactrocera ruiliensis* (Diptera: Tephritidae: Dacinae) is sequenced and annotated. The mitochondrial genome is 15,870 bp (GenBank No. MN477221) and has an A + T content of 73.3% (A 39.2%; C 16.2%; G 10.3%, and T 34.4%), which is the classical structure for insect mitogenome. All PCGs started with ATN, except *ATP8*, which is started with TTG; 12 PCGs use TAR (TAA/TAG) as the stop codon, except *COX1*, which ends with single T––. The phylogenetic tree confirms that *B. ruiliensis* clustered with other *Bactrocera* species. This study enriches the mitogenomes of the fruit flies.

The genus *Bactrocera* Macquart belongs to Dacinae (Diptera: Tephritidae). At present, more 470 species of the genus are known from tropical, subtropical, and warm temperate regions of Asia, as well as in Australia and the South Pacific, with a few species distributed in southern Africa and the Hawaiian Islands (Wang et al. 2008; Yu et al. 2014). Some species of the genus are important quarantine pests, the identification of *Bactrocera ruiliensis* is mainly based on morphology and *COI* sequences, and no mitogenome data was available. Here, we have sequenced and determined the complete mitogenome of *B. ruiliensis* using next-generation sequencing.

Total genome DNA was extracted from male adult of *B. ruiliensis* which was collected in Orchard, Huaxi District, Guiyang City, Guizhou province, China (E 106°37′11″, N 26°37′11″), in July 2018. The voucher of specimen and its genome DNA are deposited in the Institute of Entomology, Guizhou University, Guiyang, China (GUGC), accession number of them is GUGC-IDT-00125. These sequences were assembled using Geneious (v 10.2.3) (http://www.geneious.com/) (Kearse et al. 2012). Additionally, we used the MITOS server (http://mitos.bioinf.uni-leipzig.de/index.py) (Bernt et al. 2013) and tRNA scan-SE server (Lowe and Chan 2016) for annotation. The ML (maximum-likelihood) tree was constructed to investigate the molecular taxonomic position of *B. ruiliensis* using the nucleotide sequences of 13 protein-coding genes and two rRNA genes using IQ-TREE v1.6.3 (Nguyen et al. 2015).

The complete mitogenome of *B. ruiliensis* is 15,870 bp (GenBank No. MN477221), containing 13 protein-coding genes (PCGs, 11,209 bp), 22 transfer RNA genes (tRNAs, 1467 bp), two ribosomal RNA genes (rRNAs, 2142 bp), and a large non-coding region (control region, 882 bp). The overall base composition of the genome is 39.2% A, 16.2% C, 10.3% G, and 34.4% T, exhibiting an obvious A + T bias (73.3%). The AT-skew (0.070) for the whole mitogenome is slightly positive while GC-skew (−0.223) is negative. All PCGs started with ATN (ATA/ATG/ATT/ATC), except *ATP8*, which is started with TTG; 12 PCGs use TAR (TAA/TAG) as the stop codon, except *COX1*, which ends with single T––.

The phylogenetic relationships of *B. ruiliensis* were reconstructed with IQ-TREE using an ultrafast bootstrap approximation approach with 10,000 replicates based on concatenated the nucleotides of the 13 PCGs and two rRNAs with 13,197 bp (Figure 1). Each PCG and rRNA sequence was aligned using the MAFFT algorithm in TranslatorX and MAFFT v7.0 online serve with the G-INS-i strategy, respectively, and aligned sequences were eliminated using Gblocks 9.1 b (Abascal et al. 2010; Katoh et al. 2017). The phylogenetic tree confirms that *B. ruiliensis* as the sister group to *B. dorsalis*, *B. invadens, B. papaya, B. philippinensis,* and *B. carambolae*. Up to now, not a lots of studies have been recorded for *Bactrocera*, and we hope that our data can be useful for further study.

**Figure 1.**
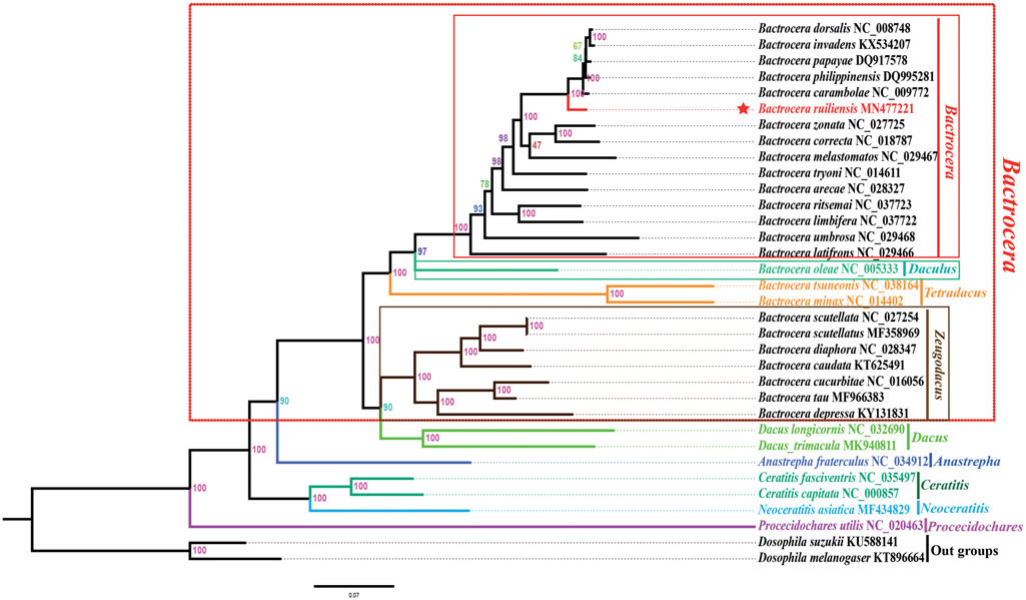
Phylogenetic analyses of *Bactrocera ruiliensis* based upon the concatenated the nucleotides of the 13 PCGs and two rRNAs of 32 ingroup species using IQ-TREE. Numbers at nodes are bootstrap values. The accession number for each species is indicated after the scientific name.
